# Supplementary material for: Lipid-mediated Wnt protein stabilization enables serum-free culture of human organ stem cells
Source: Nat Commun. 2017 Mar 6;8:14578. doi: 10.1038/ncomms14578 (PMC5343445; doi:10.1038/ncomms14578)
Supplement: Supplementary Information — Supplementary Figures, Supplementary Tables. [file ncomms14578-s1.pdf]

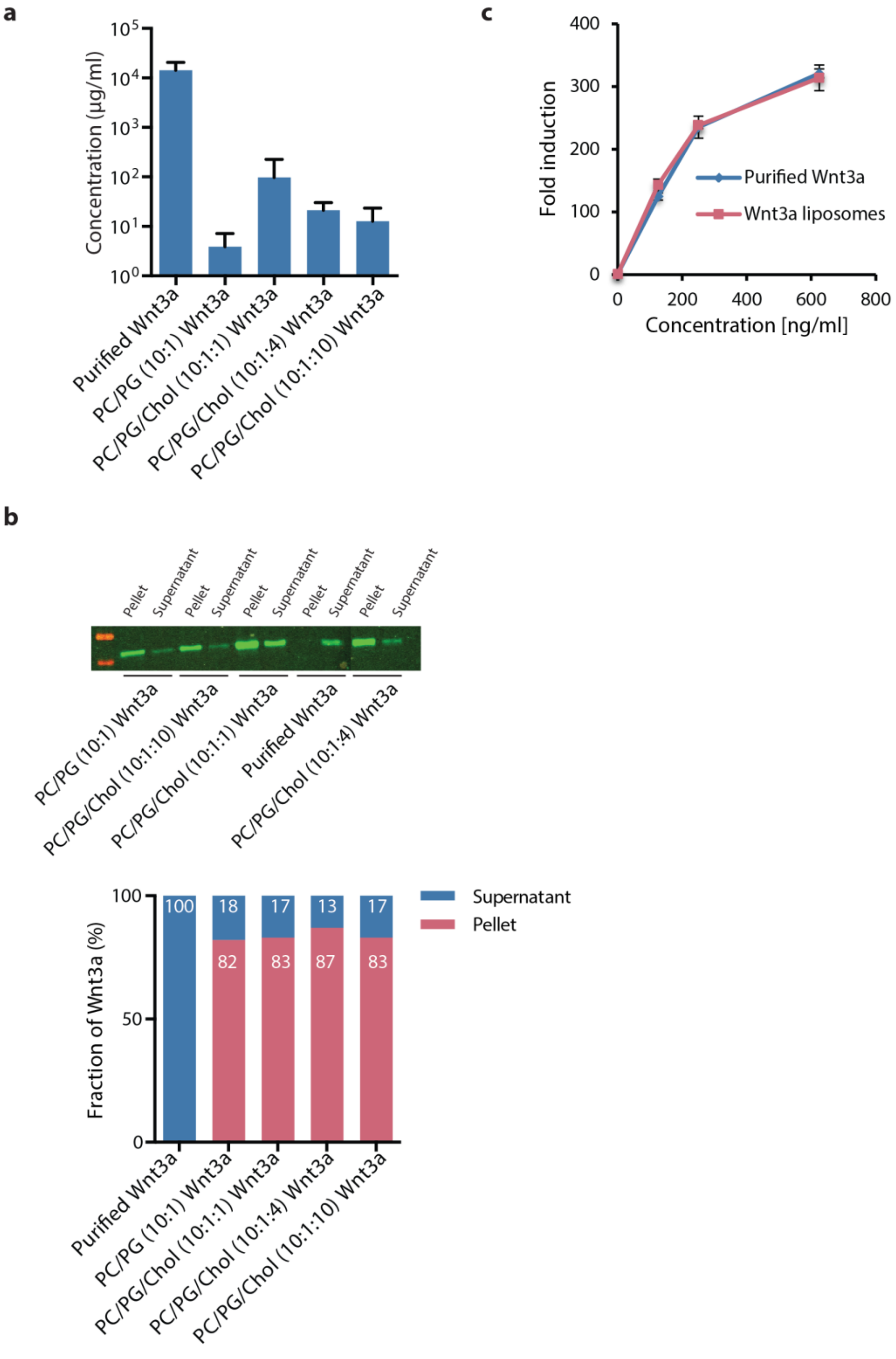

Supplementary Figure 1: Characterization of Wnt3a-liposomes. (**a**) Determination of CHAPS content by high-performance liquid chromatography in Wnt3a liposomes of various compositions after dialysis (n=3, mean±SEM). (**b**) Fraction of Wnt3a protein found in supernatant and pellet after ultracentrifugation of Wnt3a-liposomes, determined by Western blotting. (**c**) Quantification of Wnt activity immediately after addition of the indicated reagents to serum-free medium (n=3, mean±SEM).

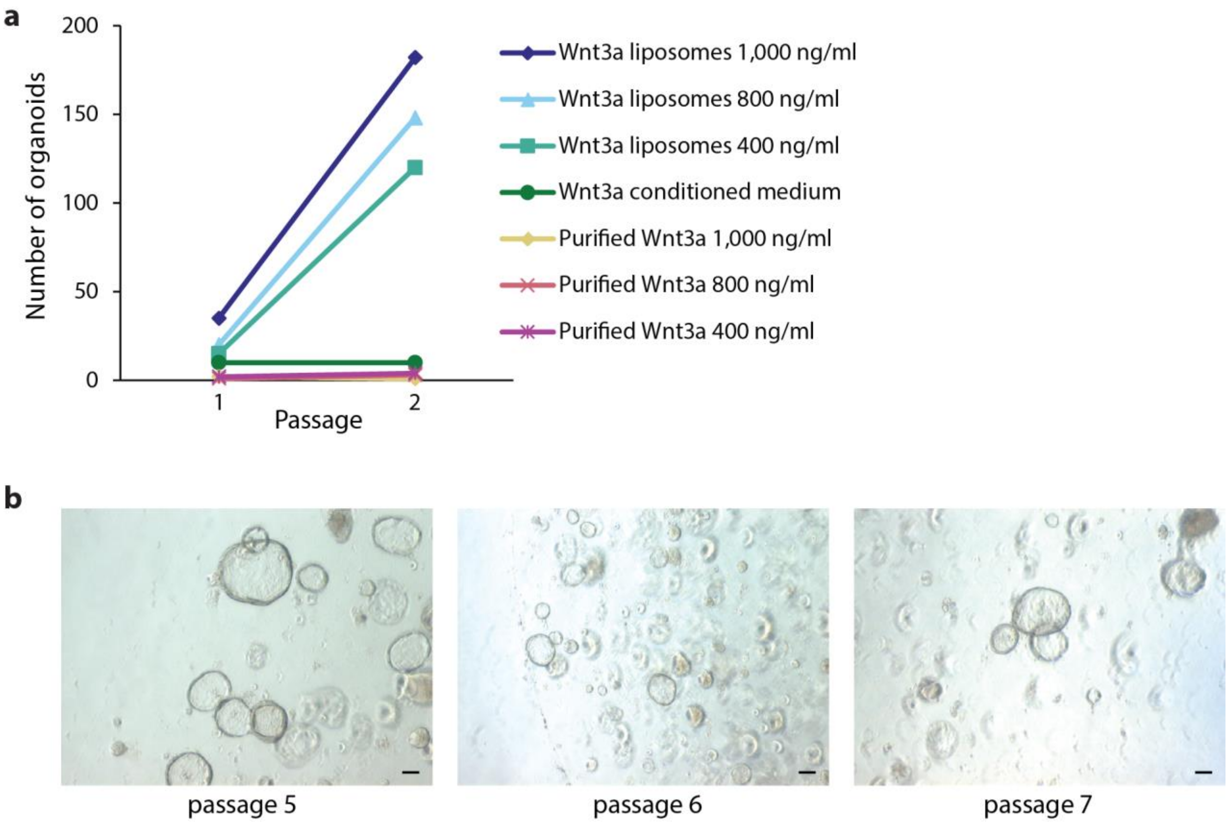

Supplementary Figure 2: Derivation and propagation of human jejunum organoid cultures. (**a**) Efficiency of derivation of human jejunum organoids (donor 2) in the indicated conditions in serum-free medium. (**b**) Human jejunum organoids derived and maintained in the presence of Wnt3a liposomes displayed robust expansion. Organoids were passaged 1:6 every 10 days and reconstituted a similar density of organoids following each passage. Scale bars 100 µm.

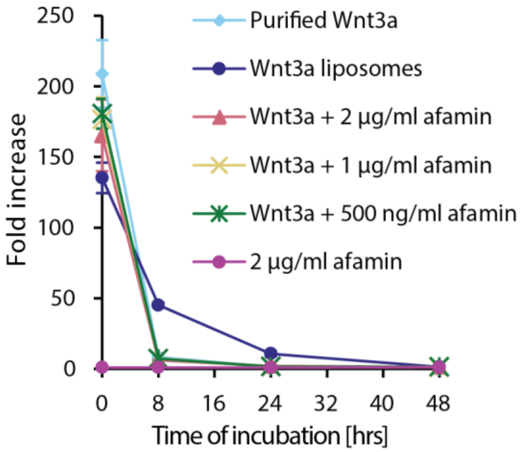

Supplementary Figure 3: Effect of recombinant afamin on Wnt3a stability. Quantification of Wnt activity retained after incubation of the indicated reagents (containing 250 ng/ml Wnt3a where indicated) in serum-free medium for the indicated amounts of time at 37°C (n=3, mean±SEM).

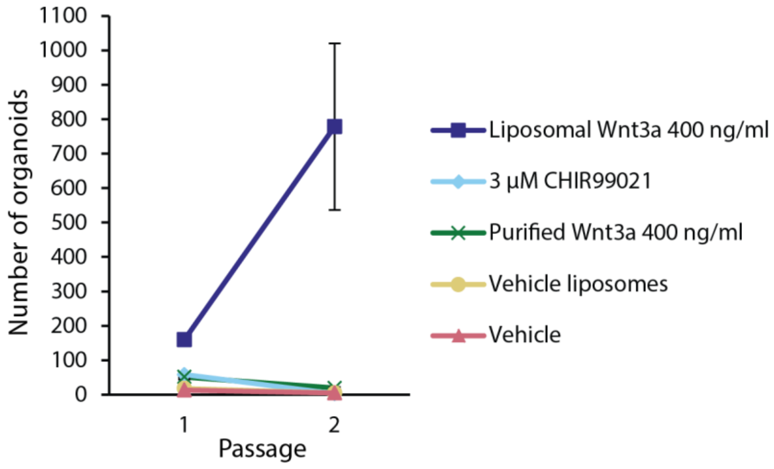

Supplementary Figure 4: Effect of the GSK3 inhibitor CHIR99021 on human duodenum organoid expansion. Expansion of single human duodenum organoid cells (passage 12) able to form new organoids in the indicated conditions (n=3, mean±SEM).

Supplementary Table 1. Physical properties of liposomes determined by dynamic light scattering.

Type of liposome (molar ratio)	Mean diameter (nm) ± S.D. (n=2)	Polydispersity index (0-1) ± S.D. (n=2)
DMPC/DMPG (10:1)	142 ± 5	0.14 ± 0.01
DMPC/DMPG/Cholesterol (10:1:1)	152 ± 11	0.20 ± 0.12
DMPC/DMPG/Cholesterol (10:1:4)	144 ± 16	0.06 ± 0.00
DMPC/DMPG/Cholesterol (10:1:10)	130 ± 1	0.04 ± 0.02
Dialyzed DMPC/DMPG (10:1) + Wnt3a	208 ± 74	0.25 ± 0.29
Dialyzed DMPC/DMPG/Cholesterol (10:1:1) + Wnt3a	206 ± 159	0.31 ± 0.20
Dialyzed DMPC/DMPG/Cholesterol (10:1:4) + Wnt3a	164 ± 10	0.16 ± 0.07
Dialyzed DMPC/DMPG/Cholesterol (10:1:10) + Wnt3a	132 ± 1	0.14 ± 0.05

Supplementary Table 2. Lipid vesicles maintain their physical characteristics upon reconstitution following lyophilisation.

Type of liposome	Mean diameter (nm) ± S.D. (n=3)	Polydispersity index (0-1) ± S.D. (n=3)
Wnt3a liposomes (before lyophilisation)	123 ± 1	0.03 ± 0.02
Vehicle liposomes (before lyophilisation)	126 ± 3	0.06 ± 0.03
Wnt3a liposomes (reconstituted)	133 ± 2	0.09 ± 0.10
Vehicle liposomes (reconstituted)	124 ± 15	0.07 ± 0.03
